# Annotations

**Published:** 1903-08-22

**Authors:** 


### ANNOTATIONS.

### Rowdyism *versus* Education.

The prorogation of Parliament has, it may be hoped, silenced for a time the advocacy, by law-abiding people, of the organised rowdyism which its supporters, oddly enough, have thought fit to describe as "passive resistance" to the education rate. It is difficult not to feel some sorrow for a member of the House of Commons who fancies himself compelled, by the operation of party currents in his constituency, to endeavour to find excuses for persons who deliberately break the law which the House has as deliberately enacted, and who thereby strike at the very root of the principles on which representative government is based. It must, we think, be admitted that it is on the whole better to endeavour to improve the unenlightened "conscience" than to ask the more intelligent members of the community to bow to its supposed dictates; and, if any evidence of the necessity for an improved education law were required, it would be abundantly furnished by recent evidence of the manner in which the more ignorant of the lower classes are being manipulated for party purposes by rowdy preachers and by the baser sort of hired politicians. The case, as it now stands, is eminently one for Government, that is to say, for the strict enforcement, by the properly constituted authorities, of the will of the people as it has been emphatically expressed through the great majority of their representatives in Parliament. Conscience, like other faculties, needs to be instructed; and the channels into which the public conscience, in the present state of our civilisation, has most need to be directed, are those which are conducive to clean living, and to orderly behaviour. If these can be promoted or secured, a goodly number of other questions, not necessarily unimportant, may be safely left to take care of themselves.

### The Common House-fly.

Mankind, up till quite recently, has been disposed to regard the common house-fly as a friend rather than an enemy, and has credited it with the virtues of a harmless, painstaking, and efficient scavenger. During the last few years, however, our notions have undergone a complete change, and it has been demonstrated that as a carrier of disease organisms the house-fly is one of our worst enemies. This will be readily believed when it is remembered that the insect breeds in horse manure and the excreta of other animals, which are therefore its favourite resorts. It is obviously disgusting to allow such a creature to settle upon our food before we eat it ourselves. The connection of the fly with enteric fever was brought out very forcibly during the Hispano-American war. Dr. Vaughan, who was one of the commissioners appointed to investigate into the prevalence of enteric fever in the United States Army during the summer of 1898, reported that flies swarmed over infected fæcal matter in the pits, and then visited and fed upon the food prepared for the soldiers at the mess tents. In some instances where lime had recently been sprinkled over the contents of the pits, flies, with their feet whitened with lime, were seen walking over the food. Another disease, the annual mortality from which in the United Kingdom alone is very great, is the summer diarrhœa of children. The cause and possibility of prevention of this disorder are receiving a good deal of attention just now from public health officials, and here again something more than a mere suspicion has been directed towards *musca domestica*. It has been suggested that the fly also plays an important *rôle* in conveying the infection of cholera, carbuncle, ophthalmia, and other diseases, and the suggestion will probably be found to be true in part at least. A fly's leg, covered as it is with "hairs," and provided with a sucker foot, is just the thing for conveying fine particles of dirt and micro-organisms from one habitat to another. In any case these illustrations should be a sufficient warning, and although it may not be practicable to exclude the insect altogether from human habitations and human food, yet it is as well to appreciate that the insect has great potentialities for evil.

### Disinfection Tests.

There are a good many people who are inclined to place little faith on methods of disinfection, and their scepticism is probably due to the fact that in the first place the process is often carelessly performed, and in the second because there is not often to be had an obvious proof that much has been achieved. The proof that the sulphur candle, or whatever else may have been employed, has done its work is frequently hard to find. M. Calmette has, however, invented tests for sulphur and formaldehyde, quite simple in themselves, but capable of showing whether or not the agent employed has been effective. The apparatus for testing is by no means elaborate, and consists of glass tubes about a metre in length, closed at one end, and filled with fine sand, which has been coloured blue by means of tincture of sunflower. The action of sulphur is to turn this substance red, and the test for the activity of the sulphur used in disinfecting the room is simply to place the tubes in the room while disinfecting is going on. The depth to which the sand turns red is the proof of the comparative efficacy of the sulphur. The formaldehyde test is similar in process but different in material. In this case the tubes are filled with undyed sand, into which are introduced small particles of dried serum which has been coloured red by being mingled with a solution of fuchsine rubine. The stain, at the time the serum is put in the tubes, is easily dissolved out in water, and colours it in dissolving. But the action of formaldehyde is to fix the colour and make it insoluble. It is therefore easy, by putting the particles of serum in water and observing their behaviour, to judge to what extent the gas has penetrated. In addition to these chemical tests, M. Calmette employs a bacteriological one. He places in open glass cylinders slips of paper which have been impregnated with the bacteria which it is sought to destroy. After keeping these tubes at a temperature of 30° Centigrade for 24 hours, some of them are placed in the room to be disinfected, and the remainder kept as checks upon these. After the disinfecting process all are placed in nutritive bouillon, and then kept in the incubator for 48 hours. The sufficiency of the disinfection is decided by whether or not the tubes which were in the infected room while the experiment was performed are found to be sterile.

